# Strut protrusion and shape impact on endothelial shear stress: insights from pre-clinical study comparing Mirage and Absorb bioresorbable scaffolds

**DOI:** 10.1007/s10554-017-1124-0

**Published:** 2017-04-01

**Authors:** Erhan Tenekecioglu, Yohei Sotomi, Ryo Torii, Christos Bourantas, Yosuke Miyazaki, Carlos Collet, Tom Crake, Solomon Su, Yoshinobu Onuma, Patrick W. Serruys

**Affiliations:** 1 Department of Interventional Cardiology, Erasmus University Medical Center, Thoraxcenter, Rotterdam, The Netherlands; 2 Department of Cardiology, Academic Medical Center, University of Amsterdam, Amsterdam, The Netherlands; 3 Department of Mechanical Engineering, University College London, London, UK; 4 Department of Cardiology, University College of London Hospitals, London, UK; 5 Manli Cardiology, Singapore, Singapore; 6 Imperial College, London, UK; 7 Emeritus Professor of Medicine Erasmus University, Westblaak 98, 3012KM, Rotterdam, The Netherlands

**Keywords:** Protrusion, Strut geometry, Shear stress, Bioresorbable scaffold

## Abstract

Protrusion of scaffold struts is related with local coronary flow dynamics that can promote scaffold restenosis and thrombosis. That fact has prompted us to investigate in vivo the protrusion status of different types of scaffolds and their relationship with endothelial shear stress (ESS) distributions. Six Absorb everolimus-eluting Bioresorbable Vascular Scaffolds (Absorb, Abbott Vascular) and 11 Mirage sirolimus-eluting Bioresorbable Microfiber Scaffolds (Mirage, Manli Cardiology) were implanted in coronaries of eight mini pigs. Optical coherence tomography (OCT) was performed post-scaffold implantation and obtained images were fused with angiographic data to reconstruct the three dimensional coronary anatomy. Blood flow simulation was performed and ESS distribution was estimated for each scaffold. Protrusion distance was estimated using a dedicated software. Correlation between OCT-derived protrusion and ESS distribution was assessed for both scaffold groups. A significant difference was observed in the protrusion distances (156 ± 137 µm for Absorb, 139 ± 153 µm for Mirage; p = 0.035), whereas difference remained after adjusting the protrusion distances according to the luminal areas. Strut protrusion of Absorb is inversely correlated with ESS (r = −0.369, p < 0.0001), whereas in Mirage protrusion was positively correlated with EES (r = 0.192, p < 0.0001). Protrusion distance was higher in Absorb than in Mirage. The protrusion of the thick quadratic struts of Absorb has a tendency to lower shear stress in the close vicinity of struts. However, circular shape of the less thick struts of Mirage didn’t show this trend in creating zone of recirculation around the struts. Strut geometry has different effect on the relationship between protrusion and shear stress in Absorb and Mirage scaffolds.

## Introduction

A permanent metallic stent constitutes a foreign structure in the vessel wall that induces inflammatory reactions increasing the thrombosis risk at post-implantation follow up [1]. Bioresorbable scaffolds (BRS) have been introduced to address the disadvantages of metallic drug-eluting stents (DES) [2]. Despite its potential long term superiorities, BRS implantation requires some indispensable rules for the attention of good stent result at short and long term follow up [3]. Embedment of the stent/scaffold is one of the surrogate factor affecting the vessel wall reaction after implantation [4]. In metallic stents, the vessel wall stretch and injury have been shown to be related with the embedment status of the device [5, 6]. Not only the embedment, but also the strut protrusion and related local coronary flow hemodynamics, particularly endothelial shear stress (ESS) influence biological response of the vessel wall [7, 8]. Observational atherosclerosis studies have proposed that low ESS promotes atherosclerotic plaque growth in native vessel segments [9, 10] and neointimal hyperplasia in scaffolded segments after percutaneous coronary intervention (PCI) [8].

Optical coherence tomography (OCT) provides high resolution data and detailed insights compared to the intravascular ultrasound [11]. OCT-based computational fluid dynamic (CFD) studies enable to unravel the effects of stent/scaffold design on the local coronary flow behaviors at the strut levels [12]. We investigated the relationship between struts protrusion and local shear stress distributions in the vessel segments implanted with Absorb and Mirage.

## Method

### Study population

Eight Yucatan mini pigs with healthy coronaries underwent PCI in the three epicardial coronary arteries through the femoral access according to the standard procedures [13]. Six coronary arteries were implanted with a single Absorb everolimus-eluting Bioresorbable Vascular Scaffolds (Absorb BVS, Abbott Vascular), six coronaries with a Mirage sirolimus-eluting Bioresorbable Microfiber Scaffold (Mirage BRMS, Manli Cardiology) with 150 micron (µm) strut thickness (Mirage-150) and five coronary arteries with a Mirage BRMS with 125 µm strut thickness (Mirage-125). The protocol approval for the animal study was received from the Institutional Animal Care and Use Committee. The study was conducted in accordance to the American Heart Association guidelines for preclinical research and the Guide for the Care and Use of Laboratory Animals [14].

### Device description

**Fig. 1:**
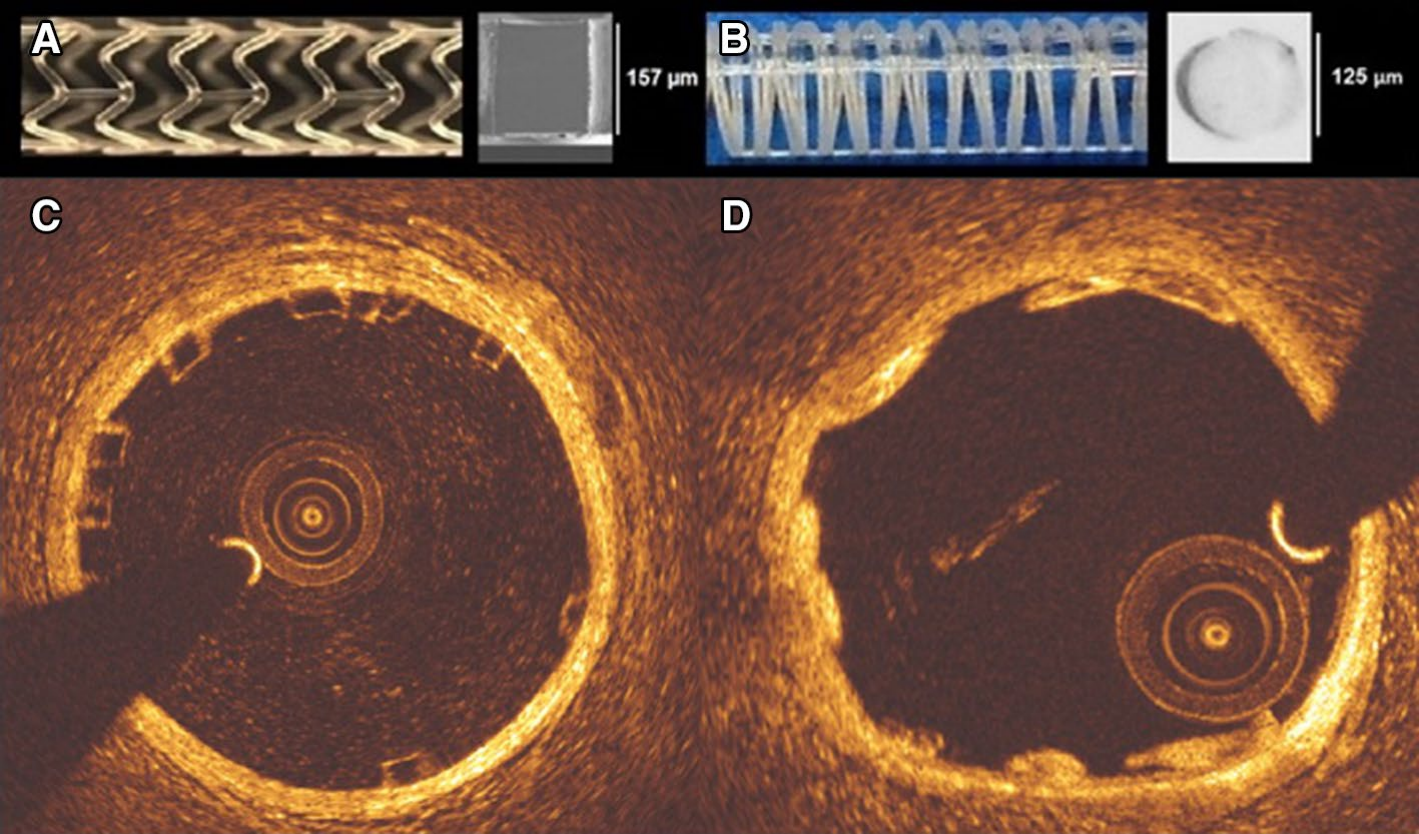
Absorb BVS 1.1 and the cross-section of Absorb BVS strut (*a*). Mirage BRMS and the cross-section of Mirage BRMS strut (*b*). While the struts of Absorb are translucent (*c*), in Mirage the struts are opaque (*d*) in OCT

Absorb BVS is composed of polymer backbone of poly l-lactic acid (PLLA), coated with a layer of a 1:1 mixture of poly d, l-lactic acid (PDLLA) eluting an anti-proliferative drug, everolimus (100 µ/cm^2^). Polymer tubes are extruded into thick-walled, small diameter tubes followed by laser cutting [15]. Absorb BVS has 157 µm strut thickness and design of in-phase zig-zag hoops linked with bridges (Fig. 1).

Mirage BRMS is made of PDLLA of which d (dextro-rotary)-isomer constitutes <5% of the total polylactic acid (PLA), coated with a biodegradable PLA delivering sirolimus. In the manufacturing process of Mirage scaffold, the polylactide monofilaments are winded and the helix-coiled structures are attached by longitudinal spine microfibers (Fig. 1). The struts of Mirage have circular shape with thickness of 125 µm in scaffolds with diameter ≤3 mm (Mirage-125), and 150 µm in scaffolds with diameter ≥3.5 mm (Mirage-150). The helicoidal design is tightened by longitudinal spine microfibers providing radial strength for the Mirage (148.54 kPa) comparable to the Absorb (148.00 kPa) [16].

### Data acquisition

Beside coronary angiography, OCT imaging was implemented after scaffold implantations in all the treated coronary arteries. The treated coronary arteries were evaluated with a frequency-domain OCT system (C8-XR OCT Intravascular Imaging System; St. Jude Medical, St. Paul, MN, USA) with a pull-back speed of 18 mm/s. A non-occlusive flushing technique was utilized by injection of the contrast media for blood clearance. The OCT analysis was performed in the scaffolded segment at every 100 µm and in the non-scaffolded segments at every 400 µm longitudinal intervals.

Coronary angiograms were analyzed with a semi-automated edge contour detection computer analysis system (CAAS QCA-2D system, Pie Medical Imaging BV, Maastricht, The Netherlands) with the dye-filled catheter used for calibration following the procedure. In each scaffold, the largest balloon diameter and maximal inflation pressure during post-dilatation were recorded and used to calculate the balloon/artery ratio (mean inflated balloon diameter/mean reference vessel diameter).

### Protrusion analysis by optical coherence tomography

**Fig. 2:**
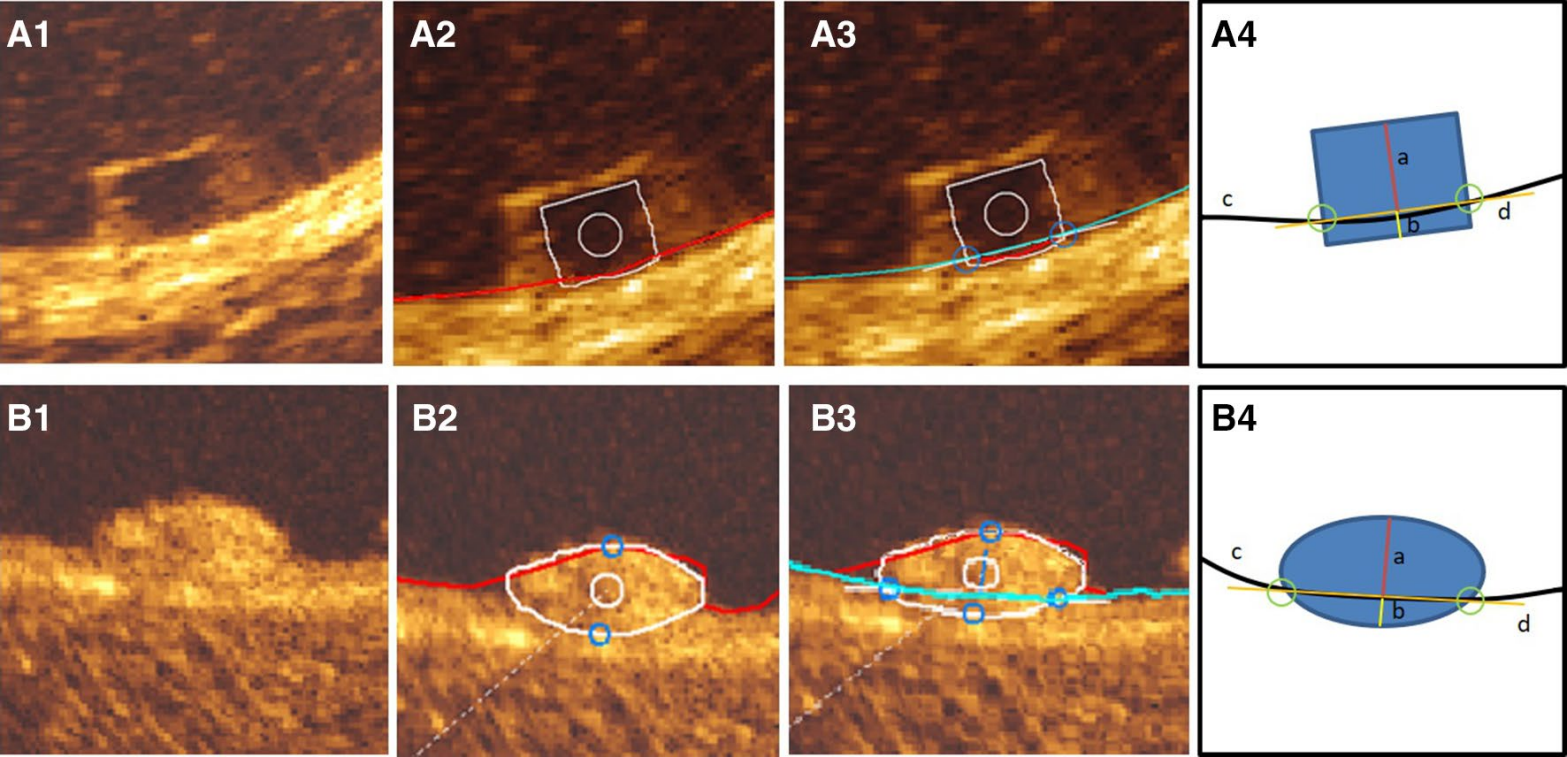
*Rectangular shaped* struts of Absorb BVS (**A1**) was automatically detected by QCU-CMS (v.14.9) after automatic detection of luminal contour (*red* contour) (**A2**) and interpolated luminal contour (*blue* contour) (**A3**), protrusion/embedment distances were detected using the methodology described by Sotomi et al. [20] (**A3**). The illustration (**A4**) demonstrates the protrusion distance (*a*), embedment depth (*b*), interpolated luminal contour (*c*) and embedment line (*d*). The adluminal surface of the *circular* struts of Mirage BRMS (**B1**) were detected during automatic detection of luminal contour (*red*) (**B2**). After automatic detection of interpolated luminal contour (*blue* contour) (**B3**) protrusion distances were measured automatically by the software (**B3**). The illustration (**B4**) demonstrates the protrusion distance (*a*), embedment depth (*b*), interpolated luminal contour (*c*) and embedment line (*d*)

For protrusion analysis, the protrusion distances were estimated semi-automatically using a special version of QCU-CMS software (version 4.69, Leiden University Medical Center, Leiden, The Netherlands) [17] (Fig. 2). The protrusion analysis in OCT was performed in the scaffolded segment at every 200 µm longitudinal interval using a methodology presented previously [17]. The luminal borders of both scaffold types and each Absorb BVS strut was automatically detected by the software. Since the QCU-CMS has no automatic detection function for the circular struts of Mirage BRMS, the struts of the Mirage were depicted as part of the lumen contours. Secondarily, the interpolation of the true lumen contour allows an interpolation that detects the protrusion of the Mirage struts. In Absorb and Mirage groups, struts located at a side branch ostium were excluded from the protrusion analysis. Since, at hinge points the maximum width of the strut in Absorb is 800 µm, the struts with >800 µm in Mirage were excluded from the protrusion analysis [4].

For the assessment of the intra- and inter-observer reproducibility, two analysts (Observer-A, ET, and observer-B, YM) performed OCT protrusion analysis. For the intra-observer reproducibility, observer-A repeated the measurements on the same pullback for 100 struts after an interval of 4-weeks. For the inter-observer reproducibility, the analysis was repeated in 100 struts by observer-B.

### Coronary artery reconstruction

Coronary artery reconstruction was implemented using a validated methodology [18]. In X-ray angiographic and OCT images, the radiopaque markers and the anatomical landmarks (i.e. side branches), identified both on angiography and on OCT, were used to define the scaffolded segment and proximal–distal native vessel segments. In the region of interest (ROI), the OCT images depicting the scaffolded and the proximal–distal native vessel segments were identified and analyzed at a 0.1 mm interval in the scaffolded segment and 0.4 mm interval in the native vessel segments. The flow area was delineated and defined in the native segments by the luminal border and in the scaffolded segments by the adluminal side of the struts and by the luminal surface borders between the struts.

Two post-procedure end-diastolic angiographic images with at least 30º-angle difference illustrating the ROI with minimal foreshortening were selected with the table in the isocenter. In these images, the luminal borders were portrayed for the ROI and used to extract the luminal centerline which was then used for the three-dimensional (3D) luminal centerline of the ROI [18]. The borders of flow area identified on OCT images were then mounted perpendicularly onto the luminal centerline and side-branches seen in both OCT and angiographic images were utilized to establish the absolute orientation of the OCT frames [18].

### Computational flow dynamic study

The reconstructed images were processed with CFD techniques. A finite volume mesh was generated and then blood flow simulation was performed. ESS was estimated by solving the 3D Navier–Stokes equations (ANSYS Fluent, Canonsburg, Pennsylvania) [7]. To assess the effect of scaffold designs on the local hemodynamic micro-environment, the mesh density around the struts and within the boundary layer of the flow field between the struts was increased to have an average element edge of 30 μm (equal to 1/4 of the strut thickness). Blood was assumed to be a homogeneous, Newtonian fluid with a viscosity of 0.0035 Pa.s and a density of 1050 kg/m^3^. A steady flow profile was simulated at the inflow of the 3D models. Blood flow for each reconstruction was estimated by measuring, in the two angiographic projections, the number of frames required for the contrast agent to pass from the inlet to the outlet of the reconstructed segment, the volume of the reconstructed segment and the cine frame rate [7]. The arterial wall was considered to be rigid. No-slip conditions were imposed at the scaffold surface. At the outlet of the model zero pressure conditions were imposed. ESS at luminal surface was calculated as the product of blood viscosity and the gradient of blood velocity at the wall [9]. The ESS was measured in the native and the scaffolded segment around the circumference of the lumen per 5° interval (sector) and along the axial direction per 0.2 mm interval with the use of an in-house algorithm [9]. The recirculation zones in the vicinity of the struts were quantified based on the direction of the ESS vector and the centerline vector. Areas where the ESS vector had opposite direction to the centerline vector were considered to be exposed to recirculation zones. Recirculation zone percentage was calculated as the vessel luminal surface exposed to recirculation divided by in-device vessel luminal surface area.

### Statistical analysis

Data are expressed as mean ± standard deviation or median and inter-quartile range. Normality of distribution was tested by the Kolmogorov–Smirnov test. Group means for continuous variables with normal and non-normal distributions were compared using Student’s t tests and Mann–Whitney U tests, respectively. Categorical variables were compared using the Pearson’s Chi square test or Fischer’s exact test, as appropriate. Mixed linear model was used for the comparisons of continuous variables to take into account the clustered nature of >1 scaffolds analyzed from the same animals, >1 cross-sections from the same scaffolds and >1 struts from the same cross-sections, which might result in unknown correlations among measurements within the clusters. The protrusion heights were analysed at cross-section level and device level for each scaffold type.

Reproducibility of protrusion/embedment analysis for Absorb was previously reported by Sotomi et al. [17]. In the present study, reproducibility of the protrusion analysis for Mirage and Absorb were assessed with the interclass correlation coefficient (ICC) for absolute agreement (ICCa) with its 95% confidence intervals (CI) using randomly selected 100 Mirage and 100 Absorb struts. An ICC < 0.4 indicates bad agreement, an ICC between 0.4 and 0.75 indicates moderate agreement, and ICC values >0.75 indicates good agreement [17]. Analyses were performed using the statistical analysis program SPSS V.23 (SPSS Inc., Chicago, IL).

## Results

One left anterior descending coronary artery (LAD), three left circumflex coronary arteries (LCx) and two right coronary arteries (RCA) were implanted with an Absorb. In Mirage-150 group, 2 LAD, 1 LCX and 3 RCA, in Mirage-125 group, 2 LAD, 1 LCX and 2 RCA were treated. Procedural characteristics are shown in Table 1. Device length was shorter and expected maximum device diameter was larger in Mirage than in Absorb. Although maximum post-dilatation balloon pressure was significantly higher in Mirage, the expected diameter of post-dilatation balloon was comparable in both arms. In QCA, balloon/artery ratio (1.09 ± 0.048 for Absorb vs. 1.13 ± 0.10 for Mirage; p = 0.32) and acute percent recoil (3.15 ± 1.15% for Absorb vs. 2.56 ± 2.92% for Mirage; p = 0.65) were comparable between the scaffold groups.

**Table 1:** Procedural details

Scaffold	Absorb BVS (n = 6)	Mirage BRMS (n = 11)	p
Implanted vessel			
LAD/LCx/RCA (n)	1/3/2	4/2/5	
Device			
Device nominal size (mm)	3.0 ± 0	3.18 ± 0.37	0.12
Device length (mm)	17.5 ± 1.22	14.63 ± 0.81	<0.001
Expected maximum device diameter (mm)	3.05 ± 0.12	3.43 ± 0.42	0.01
Maximum deployment pressure (atm)	7.0 ± 0	9.82 ± 4.85	0.28
Pre-dilatation			
Pre dilatation performed, n (%)	0 (0%)	0 (0%)	1.00
Post-dilatation			
Post dilatation performed, n (%)	6 (100%)	11 (100%)	1.00
Post dilatation balloon type			
Semi-compliant balloon, n (%)	0 (0%)	0 (0%)	
Non-compliant balloon, n (%)	6 (100%)	11 (100%)	1.00
Balloon nominal size (mm)	3.5 ± 0.0	3.57 ± 0.53	0.34
Maximum post-dilatation balloon pressure (atm)	8.5 ± 2.74	16.36 ± 2.34	0.001
Maximum expected post-dilatation balloon size (mm)	3.37 ± 0.093	3.65 ± 0.59	0.08

OCT results are summarized in Table 2. The analyses were performed at cross-section level (n = 1306) and device level (n = 17). In cross-section level analysis, in-device mean luminal area was significantly larger in Mirage than in Absorb (9.07 ± 2.26 vs. 7.62 ± 1.10 mm^2^; p = 0.032). The mean scaffold area was not significantly different between the study arms (8.00 ± 0.59 mm^2^ for Absorb BVS vs. 9.17 ± 2.00 mm^2^ for Mirage BRMS; p = 0.063). The mean ESS values were comparable between Absorb and Mirage at cross-section-level analyses (0.73 ± 2.19 vs. 0.93 ± 1.83 Pa, respectively; p = 0.142).

**Table 2:** OCT analyses results in scaffold groups

Scaffold	Absorb BVS	Mirage BRMS	p
	(n = 6)	(n = 11)	
Device level			
In-device mean lumen area (mm^2^)	7.77 ± 0.70	9.34 ± 2.12	0.081
Distal reference mean lumen area (mm^2^)	5.08 ± 1.31	6.81 ± 1.75	0.038
Proximal reference mean lumen area (mm^2^)	7.46 ± 2.63	6.92 ± 1.71	0.588
Mean scaffold area (mm^2^)	8.09 ± 0.63	9.40 ± 2.07	0.129
Mean ESS (Pa)	0.73 ± 0.25	0.93 ± 0.24	0.145
Embedment distance (µm)	22.4 ± 12.8	17.2 ± 13.1	0.43
Protrusion distance (µm)	156 ± 19	139 ± 21	0.035
Protrusion distance/mean lumen diameter	0.05 ± 0.40	0.04 ± 0.47	<0.0001

	Absorb BVS	Mirage BRMS	p
	(n = 636)	(n = 670)	
Cross-section level			
In-device mean lumen area (mm^2^)	7.62 ± 1.10	9.07 ± 2.26	0.032
Distal reference mean lumen area (mm^2^)	4.74 ± 1.42	6.69 ± 1.95	0.028
Proximal reference mean lumen area (mm^2^)	6.85 ± 2.20	6.77 ± 1.93	0.67
Mean scaffold area (mm^2^)	8.00 ± 0.59	9.17 ± 2.0	0.063
Mean ESS (Pa)	0.73 ± 2.19	0.93 ± 1.83	0.142
Embedment distance (µm)	22 ± 37	17 ± 33	0.45
Protrusion distance (µm)	156 ± 137	139 ± 153	0.035
Protrusion distance/mean lumen diameter	0.05 ± 0.0038	0.04 ± 0.0034	<0.0001

Data are expressed as n (%) and mean ± standard deviation

In the device level analysis, in-device mean lumen area was comparable between the scaffolds (7.77 ± 0.70 mm^2^ for Absorb vs. 9.34 ± 2.12 mm^2^ for Mirage; p = 0.081). Mean ESS was found comparable between Absorb (0.73 ± 0.25 Pa) and Mirage (0.93 ± 0.24 Pa) (p = 0.145). In Absorb (n = 6), 75.8 ± 13.0% and in Mirage (n = 11), 62.7 ± 17.6% of the scaffolded surface was exposed to a low (<1 Pa) athero-promoting ESS environment (p = 0.11).

Although numerically less percentage of recirculation area was estimated in Mirage (2.84 ± 1.61%) than in Absorb (3.26 ± 2.07%), the difference didn’t reach to statistical significance (p = 0.65).

### Protrusion analyses

There were 4591 struts in 477 cross-sections from Absorb and 3314 struts in 593 cross-sections from Mirage recruited into the protrusion analysis. There were 128 and 97 malapposed struts in Absorb and Mirage, respectively. In cross-section level analysis, mean protrusion distances were significantly different between the scaffold groups [156 ± 137 µm for Absorb, 98.97 ± 16.47% of the strut thickness in Absorb, 139 ± 153 µm for Mirage (95.63 ± 14.48% of the strut thickness in Mirage-150, 89.52 ± 17.09% of the strut thickness in Mirage-125); p = 0.035]. After adjusting the protrusion distances according to the luminal cross-section area, the difference in protrusion distances remained significant (0.050 ± 0.038 for Absorb vs. 0.041 ± 0.034 for Mirage; p < 0.0001).

**Fig. 3:**
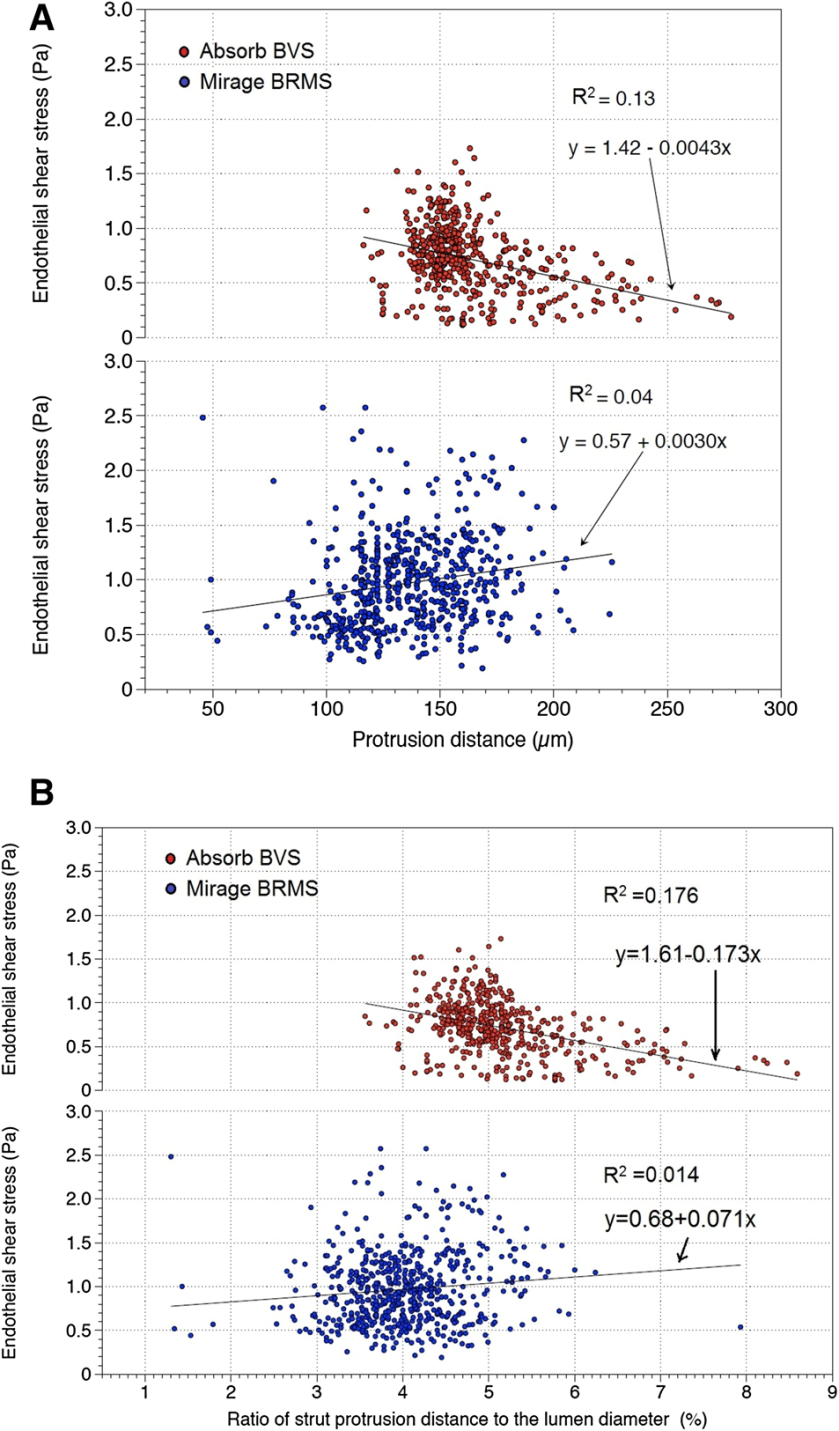
Mean shear stress values were inversely correlated with protrusion distances in Absorb BVS. However, in Mirage BRMS, shear stress values were positively correlated with protrusion distances (**a**). Mean shear stress values were in negative correlation with the adjusted strut protrusion distances in Absorb BVS. In Mirage BRMS, mean shear stress was positively correlated with adjusted protrusion distances (**b**)

Mean shear stress levels were inversely correlated with protrusion distances in Absorb (r = −0.369, p < 0.0001), whereas in Mirage, the correlation was in positive direction (r = 0.192, p < 0.0001) (Fig. 3). The same trend was observed with the adjusted protrusion distances; mean shear stress values were in negative correlation with the adjusted protrusion distances in Absorb (r = −0.420, p < 0.0001) and positively correlated with the adjusted protrusion distances in Mirage (r = 0.116, p = 0.005) (Fig. 3).

The inter-observer [ICCa: 0.884 CI (0.827–0.922)] and the intra-observer reproducibility [ICCa: 0.782 CI (0.675–0.854)] for the protrusion distances indicated good agreements in Mirage. In Absorb, the protrusion distances demonstrated well inter-observer [ICCa: 0.942 CI (0.927–0.957)] and intra-observer [ICCa: 0.968 CI (0.964–0.972)] reproducibility.

## Discussion

In the present study, we investigated the protrusion status of two different types of BRS and the effects of protrusion on the local hemodynamic microenvironment. The findings can be summarized as follows; (1) the strut protrusion distance was lower in Mirage compared to the Absorb; (2) mean ESS was comparable between Absorb and Mirage scaffolds, (3) in Mirage, ESS had a positive correlation with strut protrusion, while in Absorb, ESS was inversely correlated with strut protrusion distance.

Scaffold implantation process represents a “double-edged sword”; the struts should be embedded well enough to prevent flow separation due to the protruded obstacles in the new constituted vessel surface. There is however an increased risk of vessel wall stretch and injury in case of deep embedment [4]. The balance should be respected meticulously during the implantation process to avoid from both vessel wall injury and increment in flow disruption.

### Relationship between ESS and protrusion

**Fig. 4:**
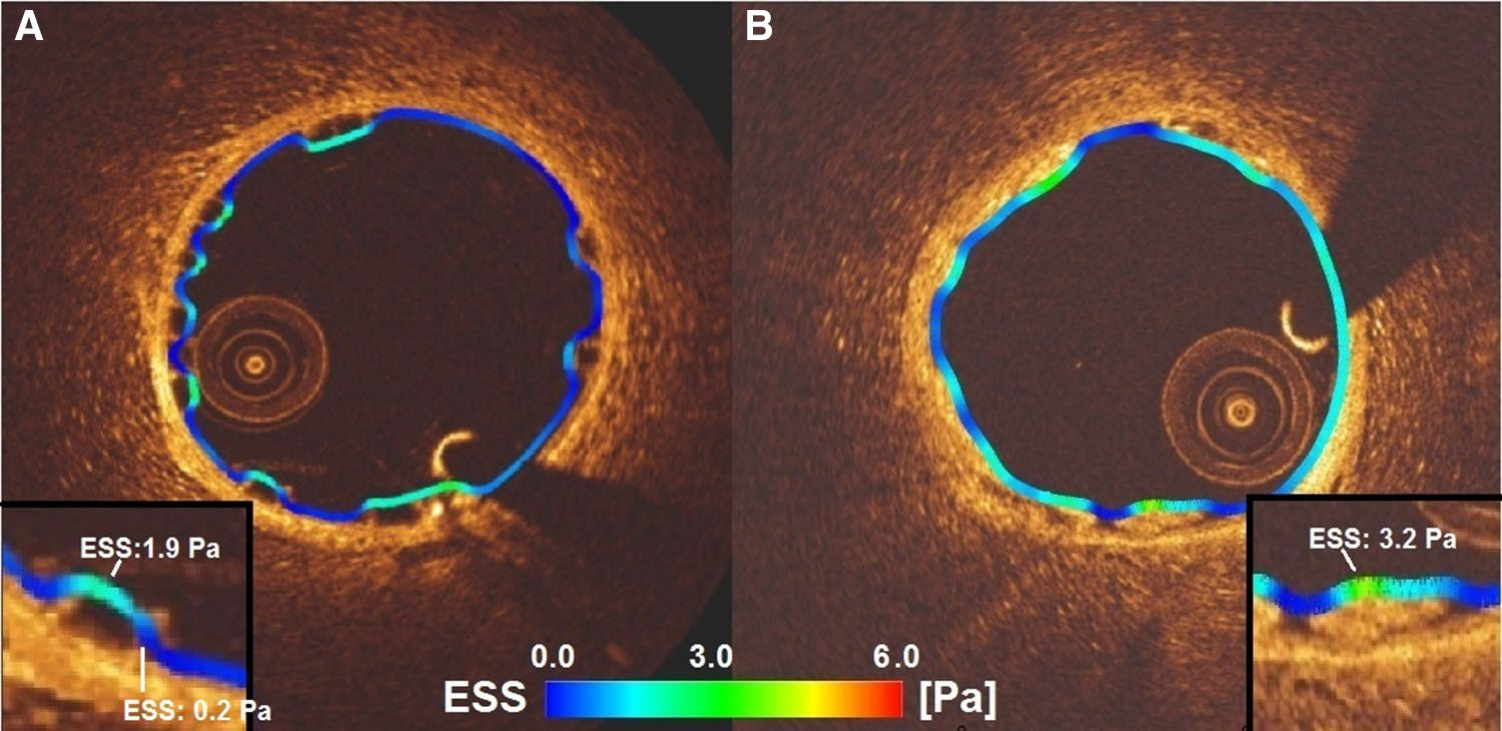
The shear stress distribution in a cross-section from Absorb (**a**) and Mirage (**b**). In Absorb, low shear stress zones can be seen between the struts wider than in Mirage. Despite comparable strut thicknesses in Absorb (157 µm) and Mirage (150 µm) the low shear stress zones (*dark blue* areas) are much less in Mirage scaffolds than in Absorb

Absorb appears to disrupt the coronary flow due to its thicker rectangular struts and high protrusion distances in the coronary lumen. Mirage, albeit lack of statistical significance, with its circular thinner struts, induced less flow separations which could be explained with the thinner streamlined strut cross-profile and the helicoidal design of the scaffold [19, 20]. Mean shear stress levels were found comparable between the scaffold groups. Despite weak correlation coefficients, ESS has a tendency to decrease with the protrusion distance in Absorb, while in Mirage shear stress is inversely proportional to the protrusion distance. This discrepancy in the relation between the protrusion and ESS in the scaffolds can be explained with the confounding factor of different strut shapes in the scaffolds. In Absorb, the non-streamlined thicker strut profile disrupts the laminar flow inducing lower shear stress around the rectangular struts due to the recirculation and stagnation zones [20]. The shear stress on top of the rectangular struts in Absorb is higher than the shear stress at upstream and downstream sides of the struts (Fig. 4). But the non-linear increase in flow disruption around the rectangular struts and related lower ESS levels should have dominated for the relation between ESS and protrusion distance in Absorb [20].

**Fig. 5:**
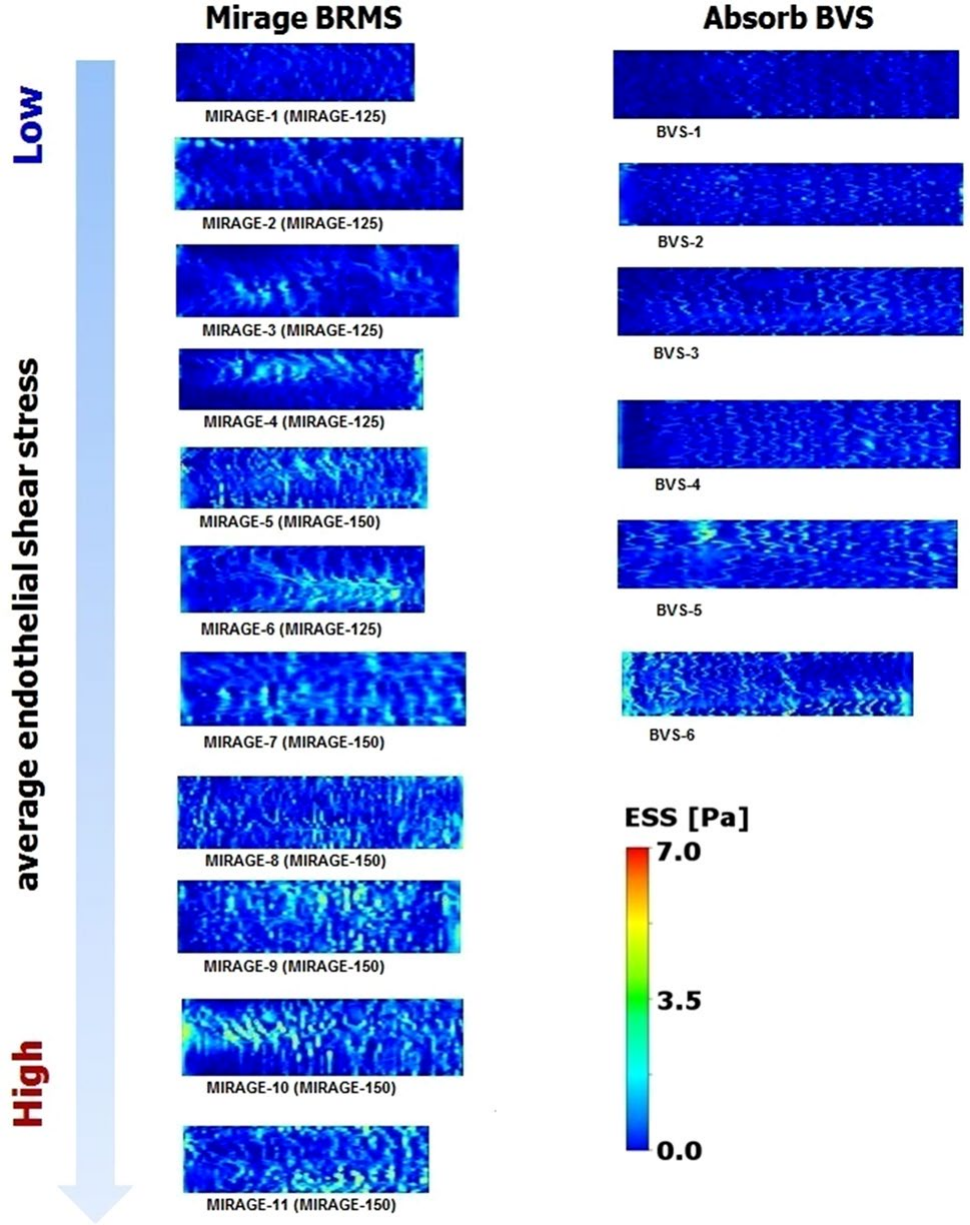
The shear stress distributions of each scaffold in carpet view. *Circular* struts demonstrate better performance than quadratic geometries in terms of shear stress distribution. The zones of shear stress within physiologic ranges seen more frequently in Mirage-150 than in Absorb BVS, despite comparable strut thicknesses

In Mirage, ESS was in positive relation with the strut protrusion which seems to be a paradox. However, reduced flow disruption due to the circular strut geometry and the gradual changes in the slope over the strut surface reversed the relation of ESS with strut protrusion [19, 20]. With the same protruded distances, circular geometries induce less flow disruptions and lower flow separation distances which provide less low shear values around the circular struts [20, 21]. Additionally, according to the Bernoulli principle, the flow accelerates over a streamlined geometry which may also help to explain the positive relation of shear stress with protrusion distance in Mirage. Accelerated flow velocity over the circular struts increases shear stress not only at the top surface but also at the upstream and downstream sides of the circular struts as the protrusion rises [22, 23] (Fig. 4). In similar strut thicknesses, circular designs demonstrate better performance in terms of shear stress levels [24]. The shear stress distribution from the present study confirms this point. In scaffolds with comparable strut thicknesses, the shear stress distributions unraveled more physiologic shear stress levels in Mirage-150 group than in Absorb (Fig. 5).

The scaffolds with similar strut profile with Absorb BVS such as DREAMS 2G and DESolve, have comparable strut thicknesses (DREAMS 2G: 150 µm [25], DESolve: 150 µm [16]). Beside the strut thickness, the width of the strut is also another factor in penetration of the strut into the vessel wall during the implantation process [26, 27]. While the width of the struts in Absorb is around 191 µm in the hoops, the struts of DREAMS and DESolve scaffolds have widths of 140 and 165 µm, which means that narrower struts can penetrate into the vessel wall deeper that corresponds to less protrusion and less flow disturbance. CFD modelings can be used to modify the strut design to reach more hemocompatible geometries.

**Fig. 6:**
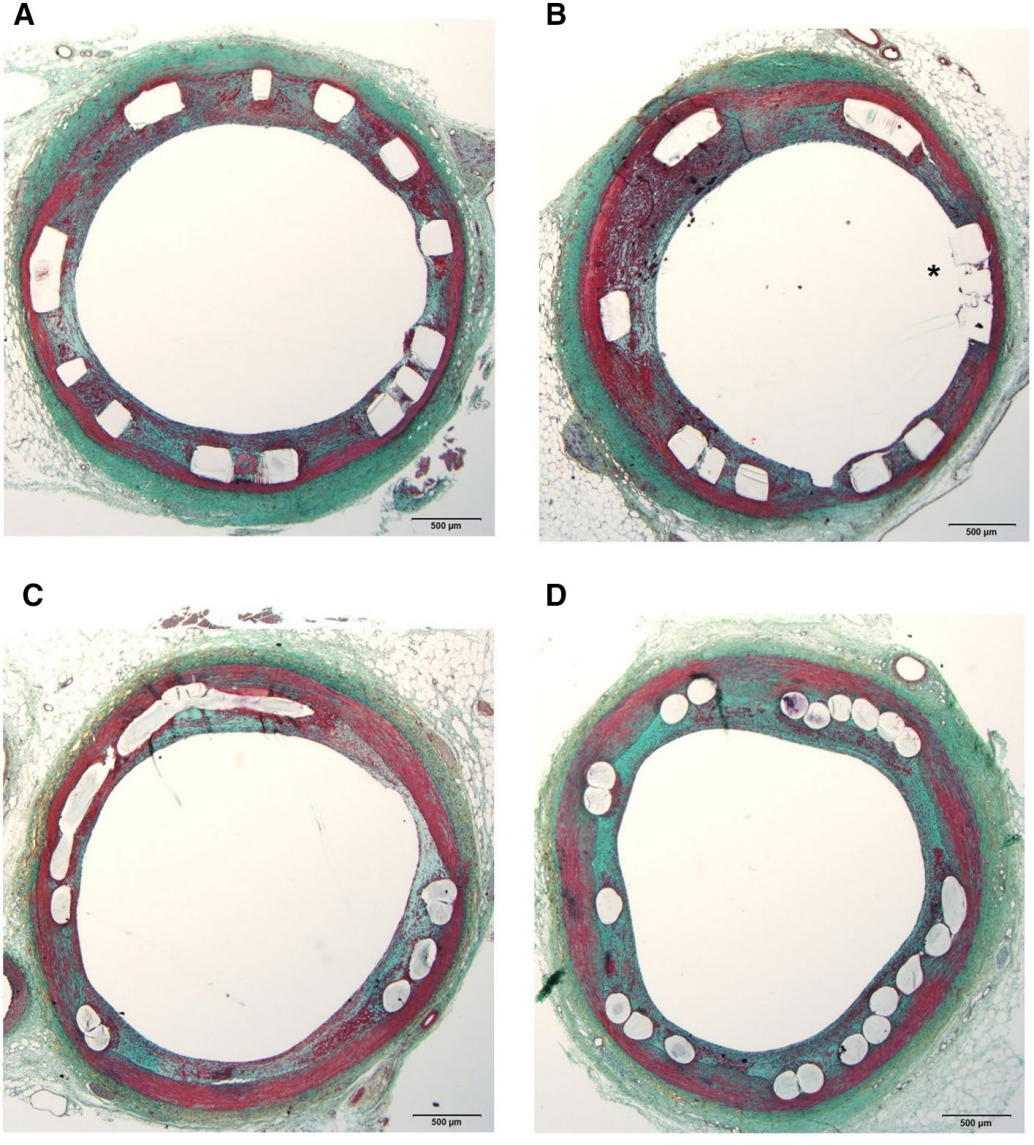
The histology sections from Absorb (**a, b**) revealed struts with insufficient endothelial coverage (*asterisk*) whereas in Mirage (**c, d**), struts were detected fully covered by neointimal tissue

Flow-disruption and decreased shear stress levels in the vicinity of the struts increase a tendency to thrombus formation which was demonstrated in bench studies [21]. Low shear stress has been incriminated for restenosis and stent/scaffold thrombosis at post-implantation follow up [28]. The flow separations beside the rectangular struts promote stagnation zones that create well-suited environment for thrombus formation. However, even with comparable aspect ratios with the quadratic geometries, the flow streamlines slipping over circular struts with less disruption decrease the risk of fibrin aggregation [21]. The fibrin accumulation and thrombus formation may affect the anti-proliferative drug kinetics. Drug diffusion from the polymer to the vessel wall can be interrupted by cumulated fibrin which may impoverish the endothelial lining through the accrued drug around the struts. Impaired endothelial sealing of the struts can increase re-stenosis risk at follow up [29]. At 30-day follow up, 2 animals from the present study sacrificed and underwent histopathological analysis. From Absorb (n = 2) and Mirage (n = 2), sections (n = 12) from proximal, middle and distal portions of each scaffold were assessed by histopathologists. The fibrin score in Absorb (2.5 ± 0.55) was higher than in Mirage (2.17 ± 0.41) (p = 0.26) and endothelial coverage rate was lower in Absorb (56.5 ± 38.07%) than in Mirage (82.33 ± 18.99%) (p = 0.18). However, due to low case number there were no statistical differences between the scaffold groups (Fig. 6).

### Limitations

A significant limitation of the current analysis is that scaffold implantation was performed in healthy coronary arteries. Therefore, it was not possible to examine the implications of scaffold under-expansion or the composition of the underlying plaque on strut embedment which potentially influence the local flow hemodynamics. There were differences in the lumen areas in the two groups which are likely to affect the ESS values. Nevertheless, the difference in ESS was not statistically significant; in addition, in linear mixed effect analysis scaffold type was independently associated with the ESS distribution. There was no clinical result for protrusion-ESS relationship in the current study. Due to low number of cases (totally 12 histology specimens from 4 scaffolds), we didn’t present histopathology results at 30-day post-implantation in detail. However, clinical inferences can be exemplified from the literature. Finally, the protrusion software used for the analysis has been established for quadratic struts of Absorb. A well-designed software for circular struts may overcome the constrain of reproducibility in Mirage scaffolds.

## Conclusion

Protrusion has impact on the local hemodynamics in bioresorbable scaffolds. Strut geometry has diverse effects on the relationship between protrusion distance and shear stress in Absorb and Mirage scaffolds. Protrusion analysis may contribute to figure out hemodynamic performance of the bioresorbable scaffolds.
